# Isoalantolactone induces intrinsic apoptosis through p53 signaling pathway in human lung squamous carcinoma cells

**DOI:** 10.1371/journal.pone.0181731

**Published:** 2017-08-04

**Authors:** Chengyan Jin, Guangxin Zhang, Yifan Zhang, Peiyan Hua, Ge Song, Mei Sun, Xin Li, Ti Tong, Bingjin Li, Xingyi Zhang

**Affiliations:** Jilin Provincial Key Laboratory on Molecular and Chemical Genetic, The Second Hospital of Jilin University, Changchun, P.R. China; Xi\'an Jiaotong University, CHINA

## Abstract

Isoalantolactone has recently been revealed to induce apoptosis in several types of cancer. However, little is reported on its anti-tumor potential on human lung cancer. Our present study was designed to investigate its effects on human lung squamous carcinoma SK-MES-1 cells. We found that Isoalantolactone induced cellular and DNA morphological changes and decreased the viability of SK-MES-1 cells. It significantly inhibited the growth of SK-MES-1 cells through apoptosis in a dose-dependent manner via activation of p53. It also induced cell cycle arrest at G1 phase. It can down-regulate Bcl-2 and up-regulate Bax, to induce dissipation of mitochondrial membrane potential and generation of reactive oxygen species. Caspase-3 was also activated by Isoalantolactone, with the cleavage of poly (ADP-ribose) polymerase. Our results reveal that Isoalantolactone induces intrinsic apoptosis in SK-MES-1 cells through p53 signaling pathway, which suggests that Isoalantolactone could be a potential leading compound for future development of anti-lung cancer drugs.

## Introduction

Lung cancer is the only malignant tumor whose morbidity and mortality rise obviously recent years. Although the treatment varies, chemotherapy still plays an important role in the first and second-line of cancer therapy. But the postoperative survival rate has no effective improvement. Studies have suggested that apoptosis is a key factor that contributes to anti-tumor therapies of chemotherapeutic drugs. Hence the development of novel agents with selectivity against critical apoptotic targets may provide a rational approach for treatment of cancers.

Herbal medicines, as an important novel source with a wide range of pharmaceutical potential, are being used for treatment of human ailments, many of which hold great potential as promising agents for treatment of cancer and have already been used in clinic [1]. Isoalantolactone, one of the major sesquiterpene lactone compounds, is isolated from the roots of Anula helenium and possesses multiple biological activities. Several studies have shown that Isoalantolactone induced apoptosis in gastric adenocarcinoma [2], prostate cancer [3], pancreatic carcinoma [4], head and neck squamous cell carcinoma [5] et al. However, the effect of Isoalantolactone on human lung cancer cells has not been reported yet.

External stimulus which could damage cells cause changes in the inner mitochondrial membrane that results in an opening of the mitochondrial permeability transition pore, loss of mitochondrial transmembrane potential and release of normally sequestered pro-apoptotic proteins from intermembrane space into cytosol, which could induce irreversible programmed cell death [6–8]. The control and regulation of these apoptotic mitochondrial events occurs through members of the Bcl-2 family of proteins. The tumor suppressor protein p53 has a critical role in regulation of the Bcl-2 family. Our present study was aimed to investigate the inhibitory effect of Isoalantolactone on human squamous carcinoma SK-MES-1 cells and possible mechanism in Isoalantolactone-induced apoptosis in lung cancer cells.

## Materials and methods

### Materials

Human lung squamous carcinoma cell line SK-MES-1 was purchased from Cell Bank of Chinese Academy of Sciences (Shanghai, China). Isoalantolactone was purchased from the Chinese materials research center (Beijing, China), dissolved in Dimethyl Sulfoxide (DMSO), which was purchased from Shenggong Company (Shanghai, China). Fetal bovine serum (FBS) was purchased from Gibco (Carlsbad, CA, USA). 3-(4,5-Dimethylthiazol-2-yl)-2,5-Diphenyltetrazolium Bromide (MTT), Hoechst 33342, Dulbecco's Modified Eagle's Medium (DMEM) and Rhodamine 123 mitochondrial specific fluorescent dye were purchased from Sigma (St. Louis, MO, USA). Cell Cycle Analysis Kit (PI+Rnase A), Annexin V-FITC Apoptosis Detection Kit, Reactive Oxygen Species Assay Kit and BCA Protein Assay Kit were purchased from Keygene Company (Nanjing, China). Polyclonal antibodies against β-actin(1:2000 dilution #4967S), Bax(1:1000 dilution #2772S), Bcl-2(1:1000 dilution #2876S), pro-caspase-3(1:1000 dilution #9662P), poly AIsoalantolactone-ribose polymerase (PARP) (1:1000 dilution #9542S), pRb(1:1000 dilution #9306S), p27(1:1000 dilution #2552), p53(1:1000 dilution #9282S), and horseradish peroxidase-conjugated secondary antibodies (goat-anti rabbit, mouse) were purchased from Cell Signaling Technology, Inc. (Shanghai, China). Western Blotting detection kit was purchased from Milipore (Billerica, MA, USA).

### Cell culture and treatments

Human lung squamous carcinoma SK-MES-1 cells were cultured in DMEM medium supplemented with 10% FBS, and maintained at 37°C with 5% CO2 in a humidified atmosphere.

### Cell growth inhibition assay

The inhibition of cell growth was determined by a MTT assay as previously described. Briefly SK-MES-1 cells were seeded in 96-well plate at density of 1×10^4^cells/well, and then treated with various concentrations of Isoalantolactone for 24 h. Following treatment, the MTT reagent was added (100 μl/ml) and cells were further incubated at 37°C for 4 h. Then 150 μl DMSO was added to dissolve the formazan crystals and absorbance was read in a micro-plate reader (Varioskan Fkash, Thermo Scientific) at 570 nm. The viable cell number was directly proportional to the production of formazan. The growth assay was repeated three times. The IC50 values were calculated using GraphPad Prism 5 (version 5.0; GraphPad Software, Inc., La Jolla, CA, USA). The percentage of inhibition was calculated as follows:

$$\text{Inhibitory ratio }\left(\text{\%}\right)=(\text{A}570_{\text{control}}-\text{A}570_{\text{sample}})/\text{A}570_{\text{control}}\times 100\text{\%}$$

### Nuclei fragmentation observed by hoechst 33342 staining

To visualize apoptotic cell death and nuclear morphology, SK-MES-1 cells were stained with Hoechst. Briefly, SK-MES-1 cells were seeded into 6-well flat bottom plate at density of 6×10^5^cells/well and treated with Isoalantolactone at the concentration of 0, 20, 40 μM respectively. After 24 h of treatment, cells were collected, washed and allowed to dry on slides. Then nuclei were stained with Hoechst for 10 min. The apoptotic cells displaying fragmented or condensed nuclei were observed under a fluorescence microscope (Model:BH2-RFL-T3, Olympus, Tokyo, Japan).

### Apoptosis analysis

SK-MES-1 cells were seeded in 6-well plate at density of 3×10^5^cells/well and treated with Isoalantolactone at the concentration of 0, 20, 40 μM respectively. After 24 h of treatment, cells were collected, washed with PBS, then the apoptotic cell death rate was examined by Annexin V-FITC and PI double staining using the Annexin V-FITC apoptosis detection kit according to the manufacturer’s instructions. After staining with Annexin V-FITC/PI, the samples were analyzed by flow cytometry (EPICS^®^ XL^™^; Beckman Coulter, Brea, CA, USA).

### Cell cycle analysis

For cell cycle analysis by flow cytometry, we used Cell Cycle Analysis Kit. Briefly, SK-MES-1 cells were seeded in 6-well plate at density of 3×10^5^cells/well and treated with Isoalantolactone at the concentration of 0, 20, 40 μM respectively. After 24 h of treatment, cells were harvested and fixed in 500 μl 70% ice cold ethanol at 4°C for 2 hours. Then, the samples were washed with PBS and incubated with RNase A and PI staining solution as the manufacturer described. After staining the samples were analyzed by flow cytometry.

### Measurement of intracellular reactive oxygen species (ROS)

Reactive oxygen species level was measured by DCF-DA detection kit according to the manufacturer’s instructions. Briefly, SK-MES-1 cells were seeded in 6-well plate at density of 3×105cells/well and treated with Isoalantolactone at the concentration of 0, 20, 40 μM respectively. After 24 h of treatment, cells were collected, washed with DMEM without FBS, and then incubated with 10mM DCF-DA at 37°C for 15 min. The stained cells were washed and re-suspended in 200 μl DMEM. The intracellular ROS mediated the oxidation of DCFH to the fluorescent compound DCF. The generation of ROS was then analyzed by flow cytometry.

### Determination of mitochondrial membrane potential (MMP)

Rhodamine 123 was used to evaluate perturbations in mitochondrial transmembrane potential in SK-MES-1 cells by flow cytometry. Briefly, SK-MES-1 cells were seeded in 6-well plate at density of 3×105cells/well and treated with Isoalantolactone at the concentration of 0, 20, 40 μM respectively. After 24 h of treatment, cells were collected, washed with PBS and then incubated with rhodamine 123 (10 μM) at 37°C for 20 min. The stained cells were washed and resuspended in 200 μl PBS. The level of mitochondrial transmembrane potential was then analyzed by flow cytometry.

### Western blot analysis

To reveal the mechanism of the apoptotic effect of Isoalantolactone, as well as the cell cycle arrest, western blot was performed for apoptosis and cell cycle related proteins. Firstly, SK-MES-1 cells were seeded in 10cm culture dish at density of 60% and treated with Isoalantolactone at the concentration of 0, 20, 40 μM respectively. After 24 h of treatment, cells were collected and washed, then the cell pellets were resuspended in lysis buffer and ultrasound lysed on ice. After centrifugation, the supernatant fluids were collected and the protein contents of the supernatant were determined using BCA Protein Assay Kit, the protein samples were stored at -20°C. Secondly, the protein lysates were separated by electrophoresis on 10% sodium dodecyl sulphate-polyacrylamide gel and transferred to a Polyvinylidene fluoride membrane (Amersham Biosciences, Piscataway, NJ). The membranes were soaked in blocking buffer (5% skimmed milk) for 2 h. To probe for all the proteins, membranes were incubated overnight at 4°C with relevant antibodies, followed by appropriate horseradish peroxidase conjugated secondary antibodies and enhanced chemiluminescence detection. We use Gel-Pro Analyzer (GelPro32, version 4.0; Media Cybernetics, Inc., Rockville, MD, USA), which could extract valuable qualitative and quantitative information from electrophoretic gels to document and store our Western blot data.

### Statistical analysis

All data represent at least 3 independent experiments and were expressed as mean ± SD. Comparisons were made using a one-way ANOVA followed by Dunnett's test. P < 0.05 was considered statistically significant.

## Results

### Isoalantolactone time and dose-dependently inhibited SK-MES-1 cells growth

**Fig 1:**
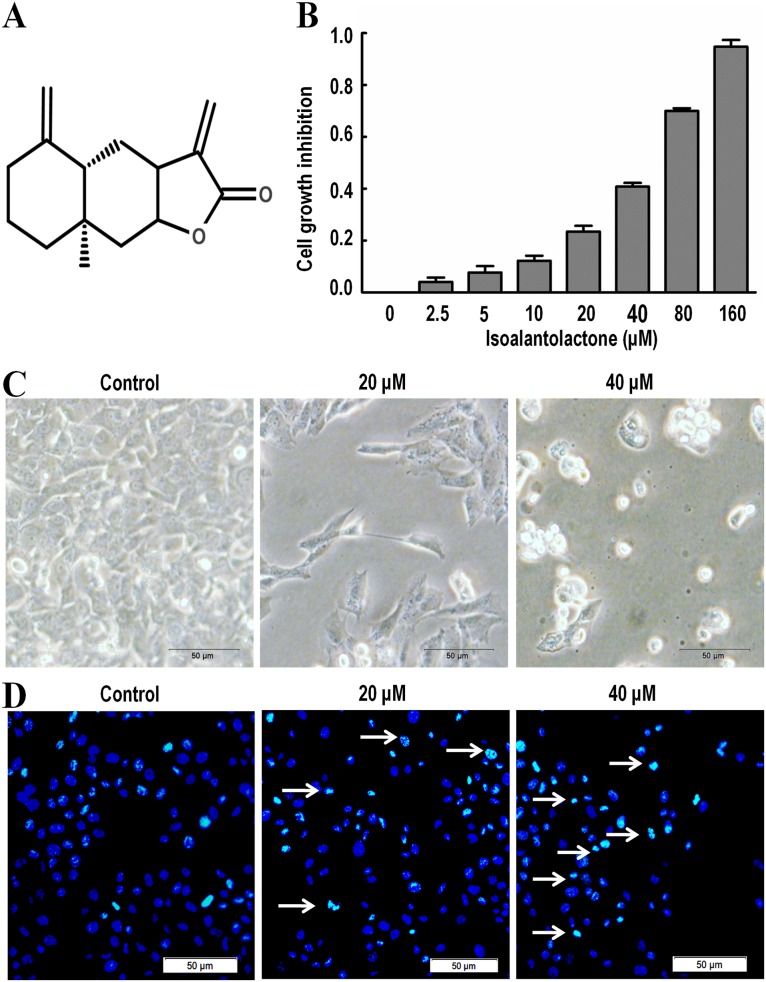
The effects of Isoalantolactone on cell viability and morpological changes in SK-MES-1 cells. (A) Chemical structures of Isoalantolactone; (B) The viability was determined by the MTT assay. Isoalantolactone expressed dose dependent inhibition effects in SK-MES-1 cells. Data are expressed as Mean ±SD of three independent experiments.; (C) Morphological changes of SK-MES-1 Cells cunder the light microscope treated with 20 and 40 μM Isoalantolactone for 24 h; (D) Nuclear morphological changes of SK-MES-1 cells using Hoechst 33342 staining and fluorescence microscopy. Treatment of cells with 20 and 40 μM of Isoalantolactone for 24 h led to a significant increase of fragmented nuclei as indicated by arrows.

Isoalantolactone (Fig 1A) isolated from the root of Inula helenium. The anti-proliferative effect of Isoalantolactone on SK-MES-1 cells was determined by the MTT assay. Treatment with Isoalantolactone for 24 h reduced the cell viability in a dose-dependent manner (Fig 1B). The IC_50_ values were about 40 μM after 24 h treatment, 20μM and 40 μM concentration after 24 h were selected for the following experiments. Morphological changes of SK-MES-1 cells after treating with 0, 20, and 40 M of Isoalantolactone for 24 h, rounded and shrunken cells and a decreased number of cells as compared to the control group (Fig 1C).

### Isoalantolactone induces apoptosis in SK-MES-1 cells

**Fig 2:**
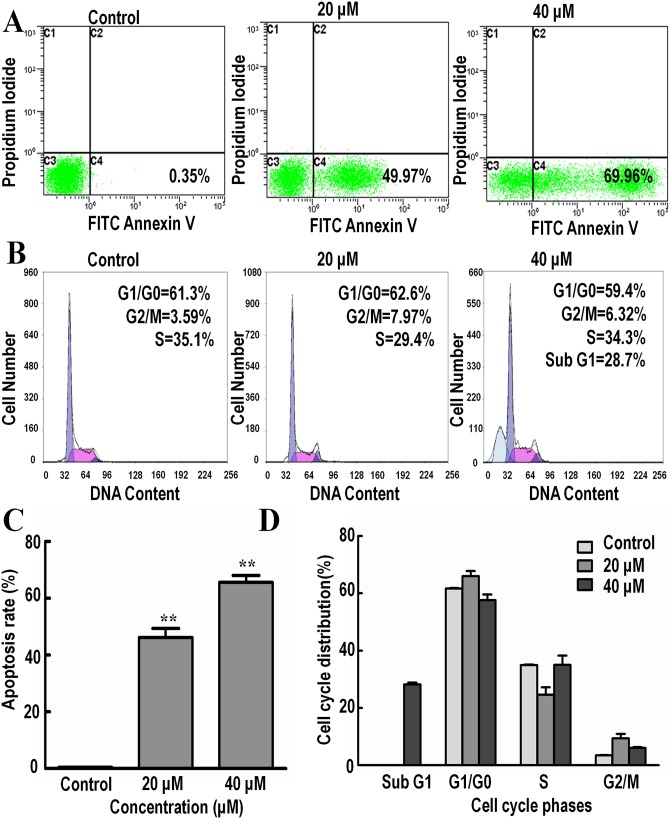
The effects of Isoalantolactone on apoptosis and cell cycle in SK-MES-1 cells. (A) Flow cytometric analysis of apoptosis with Annexin-V/PI staining. Cells were treated with different concentrations of Isoalantolactone for 24 h. The lower left quadrant (annexin V and PI negative) represents viable cells; The lower right quadrant (annexin V positive and PI negative) represents apoptotic cells in early stage; The upper left quadrant (annexin V negative and PI positive) represents necrotic cells or cellular debris; The upper right quadrant (annexin V and PI positive) represents late apoptotic or necrotic cells; Numbers refer to the percentage of apoptotic cells in early stage. (B) Flow cytometric analysis of cell cycle with PI staining. Cells were treated with 20 and 40 μM Isoalantolactone for 24 h; (C) The percentage of apoptotic cells in early stage. (D) The percentage of cell cycle distribution in different phases. Data are all expressed as Mean ± SD of three independent experiments. Columns not sharing the same superscript letter differ significantly (P < 0.05).

The effect of Isoalantolactone on cell apoptosis was analyzed by Annexin V-FITC/PI staining and Hoechst 33342 staining. DNA fragmentation is an important characteristic of apoptosis, which can be easily identified by Hoechst staining. Consistent with the previous results, treatment of cells with 20 and 40 μM of Isoalantolactone for 24 h led to a significant increase of nuclear fragmentation (Fig 1D). The results showed an increase in the percentage of dead cells in a dose-dependent manner following treatment with 0, 20 and 40 μM of Isoalantolactone for 24 h, from 0.35% to 49.97% and 69.96% (Fig 2A and 2C). The data demonstrate that Isoalantolactone induced apoptosis in SK-MES-1 cells in a dose-dependent manner.

### Isoalantolactone induces G1 phase arrest in SK-MES-1 cells

Cell cycle arrest is one of the major causes of cell growth inhibition. Induction of cell cycle arrest was analyzed using PI staining and flow cytometry analysis. Results showed that Isoalantolactone arrested the cell cycle at G1 phase in a dose-dependent manner (Fig 2B and 2D). The percentage of cells accumulated in G1/G0 phase was 61.3, 62.6 and 59.4% respectively following treatment with 0, 20 and 40 μM of Isoalantolactone for 24 h, with sub peak of G1, in part, by inducing a G1 phase cell cycle arrest.

### Isoalantolactone induces increased generation of reactive oxygen species (ROS) in SK-MES-1 cells

**Fig 3:**
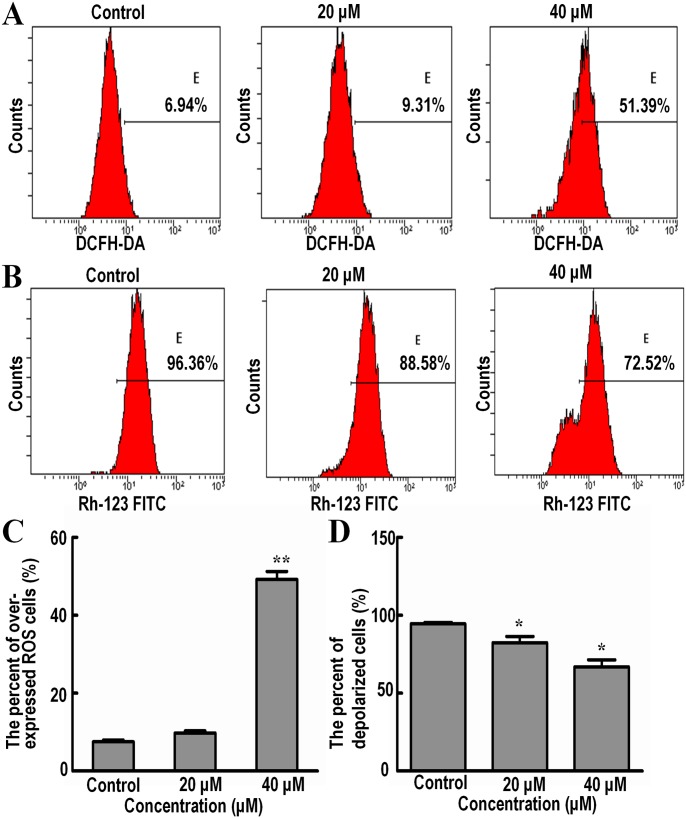
Flow cytometric analysis on ROS generation and MMP depolarization in SK-MES-1 cells treated with Isoalantolactone at 20 and 40 μM for 24 h. (A) Flow cytometric analysis of ROS stained with the fluorescent probe using DCFH-DA; (B) Flow cytometric analysis of MMP stained with the fluorescent probe Rhodamine 123; (C) Representative histograms expressed the percent of over expressed ROS cells; (D) Representative histograms expressed the percent of depolarized cells. All data are expressed as Mean ±SD of three independent experiments. Columns not sharing the same superscript letter differ significantly (P < 0.05).

Intracellular ROS generation in SK-MES-1 cells was evaluated by flow cytometry using DCFH-DA (Fig 3A and 3C), the level of ROS in SK-MES-1 cells, treated with 20 and 40 μM Isoalantolactone was significantly increased from 6.94% to 9.31% and 55.39% compared with the control group.

### Isoalantolactone causes disruption of mitochondrial membrane potential (MMP) in SK-MES-1 cells

Depolarization in MMP is a characteristic feature of apoptosis. Effects of Isoalantolactone on the MMP of SK-MES-1 cells were examined by flow cytometry using Rhodamine 123 staining. The result showed that MMP in cells treated with 20 and 40 μM of Isoalantolactone was significantly decreased from 96.36% to 88.58% and 72.52%, compared with the control group (Fig 3B and 3D). It indicates that dissipation of MMP may be one of mechanism for Isoalantolactone induced apoptosis.

### Effect of Isoalantolactone on the expression of major cell cycle and mitochondrial apoptosis regulators

**Fig 4:**
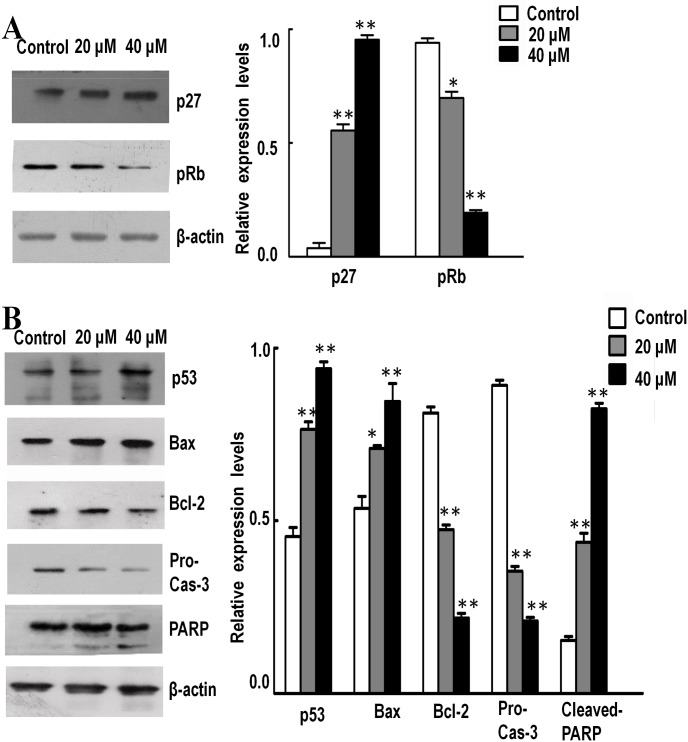
Western blot detected effects of Isoalantolactone on the expression of major cell cycle and apoptotic regulators. Gel-Pro Analyzer was performed for quantitative information. SK-MES-1 cells were treated with 20 and 40 μM of Isoalantolactone for 24 h. Following treatment, cells were harvested and total proteins were extracted as described in Materials and Methods section. (A) Treatment with Isoalantolactone increases the expression of p27 and pRb in a dose-dependent manner. (B) The expression of p53, Bax, Bcl-2, procaspase-3, and PARP were analyzed by Western blot analysis. Data are expressed as Mean ±SD of three independent experiments. Columns not sharing the same superscript letter differ significantly (P < 0.05).

To illuminate the molecular mechanism underlying G1 phase arrest mediated by Isoalantolactone, expressions of some major cell cycle regulatory proteins were detected by Western blot. The treatment with Isoalantolactone for 24 h led to up-regulation of P27 and down-regulation of phospho-retinoblastoma (pRb) in a dose-dependent manner in SK-MES-1 cells (Fig 4A).

To investigate the mitochondrial apoptosis in SK-MES-1 cells, the effect of Isoalantolactone on the expression of some major apoptosis regulatory proteins (p53, Bax, Bcl-2, Caspase-3 and PARP) were detected by Western blot (Fig 4B), the ratio for Bcl2 to Bax ratio was markedly decreased by Isoalantolactone accompanied with decreasing procaspase-3 and activation of cleavage of PARP in a dose-dependent manner. We also tested these genes expression levels by real-time PCR, as shown in Fig 4B, the mRNA expression level of p53 and Bax in SK-MES-1 cells, treated with 20 and 40 μM of Isoalantolactone were significantly increased, while mRNA expression level of Bcl-2 and Caspase-3 were significantly decreased compared with the control group, which are consistent with the results of protein expression by western blot. These results together revealed that Isoalantolactone induced apoptosis in SK-MES-1 cells through mitochondrial pathway.

## Discussion

The morbidity and mortality of lung cancer increase year after year [9, 10]. Despite given systemic therapy with surgery, chemotherapy and radiotherapy, 5 years survive rate is still not satisfied [11]. Over the last decade, many studies have revealed that herbal agents could play an anticancer role in various cancers in vitro. Isoalantolactone, as a novel agent, has been proved of capable in prevention of proliferation and induction of apoptosis in cancer cells, except lung cancer. In our study, we found that isoalantolactone exhibited negative effect on the proliferation of lung squamous carcinoma SK-MES cells in a dose dependent manner.

Cell cycle arrest and apoptosis are the major mechanisms of cell growth inhibition [12]. In our study, we found that Isoalantolactone arrested cell cycle at G1 phase. P53, a transcription factor, plays a fundamental role in the regulation of cell cycle progression, checkpoint activation, and of the apoptotic machinery in normal and cancerous cells [13–15].

Activated p53 upregulates expression of downstream target genes, including Bax and cell-cycle regulator p21, as well as p21 related protein p27, which can connected with cyclin-dependent kinases 4 and cyclin D, which leads to inhibition of phosphorylation of Rb and prevents activation of E2F-dependent transcription of genes. Rb plays a significant role during the G1/S transition, it binds to and restrains E2F family transcription factors, which regulates expression of genes necessary for cell cycle progression and prevents the cells from replicating damaged DNA [16]. Hence p27 is believed to be the inhibitor of G1 phase progression in the cell cycle. Our findings demonstrate that treatment with Isoalantolactone up-regulated levels of p27 protein and this is accompanied with obvious decrease in the levels of phosphorylated retinoblastoma protein (pRb). These data may suggest that Isoalantolactone disrupted cell cycle progression also via the activation of p53 pathway.

Previous studies have shown that Isoalantolactone inhibited abnormal proliferation by induction of apoptosis in several types of cancer cells including gastric cancer, prostate cancer, pancreatic carcinoma, head and neck squamous cell carcinoma, etc. In our study, we also found that Isoalantolactone could significently induce apoptosis in SK-MES-1 cells.

The Bcl-2 protein family is a large family of apoptosis regulating proteins that modulate the mitochondrial pathway. It includes both antiapoptotic proteins as Bcl-2 and proapoptotic proteins as Bax. These proteins regulate mitochondrial membrane permeability, either promoting or suppressing the release of apoptogenic proteins from these organelles. Several studies have indicated that p53 activates the apoptotic machinery through the regulation of Bax function and mitochondrial integrity, which results in the release of proapoptotic proteins and activation of caspases. As Bax expression increases, it is translocated to the mitochondrial membrane and increasing membrane permeability. Dysfunction of the mitochondrial membrane leads to the dissipation of mitochondrial transmembrane potential (MMP)[17], allowing the release of apoptogenic proteins from mitochondria into the cytosol, even the reactive oxygen species (ROS). Caspases play a central role in the execution of apoptosis[18]. Caspase-3 is a frequently activated death protease, which catalyzes the specific cleavage of many key cellular proteins, especially PARP [19, 20]. Our findings reveal that Isoalantolactone induced apoptosis via increasing the ratio for Bcl2 to Bax and caspase-3 with cleavage of PARP, with the dissipation of mitochondrial transmembrane potential (MMP) and increased generation of ROS.

In conclusion, our findings reveal that Isoalantolactone induced cell cycle arrest and intrinsic apoptosis in SK-MES-1 cells via activation of p53 signaling pathway. Therefore, Isoalantolactone may become a potential compound for future development of anti-lung cancer therapy.
